# Health related quality of life in older Mexican Americans with diabetes: A cross-sectional study

**DOI:** 10.1186/1477-7525-5-39

**Published:** 2007-07-12

**Authors:** James E Graham, Diane G Stoebner-May, Glenn V Ostir, Soham Al Snih, M Kristen Peek, Kyriakos Markides, Kenneth J Ottenbacher

**Affiliations:** 1 Division of Rehabilitation Sciences, University of Texas Medical Branch, Galveston, USA; 2 University of Texas-Houston Health Science Center, Houston, USA; 3 Sealy Center on Aging, University of Texas Medical Branch, Galveston, USA; 4 Department of Internal Medicine, Division of Geriatrics, University of Texas Medical Branch, Galveston, USA; 5 Department of Preventive Medicine and Community Health, University of Texas Medical Branch, Galveston, USA

## Abstract

**Background:**

The older Hispanic population of the U.S. is growing at a tremendous rate. While ethnic-related risk and complications of diabetes are widely-acknowledged for older Hispanics, less is known about how health related quality of life is affected in this population.

**Methods:**

Cross-sectional study assessing differences in health related quality of life between older Mexican Americans with and without diabetes. Participants (n = 619) from the Hispanic Established Population for the Epidemiological Study of the Elderly were interviewed in their homes. The primary measure was the Medical Outcomes Study Short Form (SF-36).

**Results:**

The sample was 59.6% female with a mean age of 78.3 (SD = 5.2) years. 31.2% (n = 193) of the participants were identified with diabetes. Individuals with diabetes had significantly (F = 19.35, p < .001) lower scores on the Physical Composite scale (mean = 37.50, SD = 12.69) of the SF-36 compared to persons without diabetes (mean = 43.04, SD = 12.22). There was no significant difference between persons with and without diabetes on the Mental Composite scale of the SF-36.

**Conclusion:**

Diabetes was associated with lower health related quality of life in older Mexican Americans. The physical components of health related quality of life uniformly differentiated those with diabetes from those without, whereas mental component scores were equivocal.

## Background

As a nation, we are growing older and more diverse. The number of adults over 65 years in 2030 will be double that of 2000 [1-5]. According to the U.S. Census Bureau, the Hispanic population increased 58% from 1990–2000 in contrast to a 3% growth in non-Hispanic Whites [6]. The older Hispanic population (≥ 65 years) is increasing at a rate double the non-Hispanic white population, and is projected to reach 15 million by 2050 [7]. Among the Hispanic population, the largest sub-group is Mexican Americans who comprise approximately 70% of the larger Hispanic population [8].

The health profiles of Mexican American older adults are known to differ from non-Hispanic Whites and include increased risk for diabetes, obesity and reduced physical activity [9-11]. In addition, Mexican American older adults may experience different social and cultural influences (i.e., diet, family networks, access to care, language barriers) that can affect their health [12]. These factors potentially impact health related quality of life in Mexican Americans in ways that are currently unknown. Qualitative studies on the cultural values and health beliefs of Hispanics with diabetes have provided no consensus as to the relative influence these characteristics can have on an individual's disease experience or management approach [13].

Significant progress has been made over the past fifteen years in examining conditions and variables that influence disability, morbidity and mortality in Mexican American older adults [14,15]. For example, Mexican American older adults with diabetes are known to be at increased risk for morbidity and mortality [16]. Mexican Americans are also at greater risk for developing complications from diabetes compared with non-Hispanic Whites [10,17]. These diabetes-related complications (e.g., nephropathy, retinopathy, neuropathy, and cardiovascular disease) lead to disability and can have negative effects on an individual's health related quality of life [18-21]. Little research has been conducted examining health related quality of life in older Mexican Americans. Previous investigations examining the relationship between diabetes and health related quality of life have been conducted with younger populations [22-24] or with non-Hispanic white older adults [18,25].

The purpose of this study was to examine differences in health related quality of life between older Mexican Americans (≥ 70 years) with and without diabetes living in the community. Our focus was on health related quality of life as defined by Guyatt and colleagues [26]. This definition does not include aspects associated with the broader concept 'quality of life' such as income, freedom, and quality of the environment. We used the *Medical Outcome Short Form *(SF-36) (see description below) which has been validated for use with Mexican American older adults [27]. Specifically, we compared scores on all eight subscales and the Physical- and Mental-Composite scales from individuals with versus without diabetes. Due to the direct relationship between diabetes and physical health (i.e. symptomology), we hypothesized that the physical component of self-reported health related quality of life would concur with the objective picture of diabetes and demonstrate significant associations.

## Methods

### Participants

The participants in the study were a sub-sample from the Hispanic Established Populations for Epidemiologic Study of the Elderly (EPESE). The Hispanic EPESE is a longitudinal study of Mexican Americans aged 65 years and older residing in Texas, New Mexico, Colorado, Arizona and California. Subjects were selected by area probability sampling procedures that involved selecting counties, and households within selected census tracts. The sampling procedure assured a sample generalizable to approximately 500,000 older Mexican Americans living in the southwest. Sampling procedures and sample characteristics have been reported previously [12,15]. The subjects were interviewed and examined in their own homes by interviewers trained by Harris Interactive, Inc., professional staff and Hispanic EPESE investigators. Training was provided on performance-based assessments of physical functioning and measurement of physical and social characteristics. The interviews were conducted in Spanish or English, depending on the respondent's preference. Baseline data were collected in 1993 from 3,050 original participants. Wave 2 interviews were conducted in 1995 with 2,439 members of the original sample (80%). 1,981 respondents were re-interviewed at Wave 3 in 1997.

*Subsample*. After Wave 3 data collection, a list of respondents who reported having Medicare coverage (n = 1,598) was created. This represented approximately 81% of the sample at Wave 3. Respondents who had Medicare coverage were chosen due to the intent of the investigators to link the sub-study data with Medicare claims data. From this list, 800 potential participants were selected using a table of random numbers to participate in a study examining the disablement process. Of the 800 respondents selected, 622 subjects participated in the sub-study. The remaining 178 respondents included those who refused to participate (n = 54) and those with proxy interviews (n = 124). We did not allow proxy interviews due to the physical nature of some measurements in the study. Two subjects died before data collection was completed resulting in a final sample for the current study of 619 participants.

### Measures of Interest

#### Diabetes

The presence of diabetes was assessed by asking the subjects whether a doctor had ever told them they had diabetes, sugar in their urine, or high blood sugar. Persons who responded positively were asked about disease duration and treatment received (categorized as none, oral hypoglycemic, insulin, or oral hypoglycemic-insulin combination). Respondents who were taking medication were asked to show the medications or prescriptions to the interviewers. 172 persons reported a medical diagnosis of diabetes with first diagnoses occurring after forty years of age. Also, included in the sample were individuals who responded in the affirmative (*n *= 20) to being told by their doctor they had borderline diabetes, sugar in urine, or high blood sugar, but were not taking any medication.

#### Health Related Quality of Life

The SF-36 is a widely used measure of self-reported health related quality of life [28]. The SF-36 consists of 36 items scored in eight scales: general health perceptions (5 items), physical functioning (10 items), role limitations due to physical functioning (4 items), bodily pain (2 items), general mental health (5 items), role limitations due to emotional problems (3 items), vitality (4 items), and social functioning (2 items). The physical functioning (PF), role limitation due to physical function (RP), bodily pain (BP), and general health (GH) scales form the basis for the Physical Composite (PCS) summary scale. The general mental health (MH), role limitations due to emotional problems (RE), social functioning (SF) and vitality (VT) ratings form the basis for the Mental Composite (MCS) summary scale. Scores range from 0 to 100, with higher values reflecting better health related quality of life [18,28,29]. The SF-36 is available in multiple languages including Spanish. Previous investigations have shown the Spanish version of the SF-36 to be a valid measure of self-reported health status for Mexican Americans as well as other Hispanic groups [27,30-32]. Cronbach's alpha for the SF-36 domains in the current study ranged from 0.76 to 0.96, suggesting good internal consistency.

#### Covariates

The ensuing list of covariates were chosen for two reasons; they are known risk factors for older Mexican Americans and/or individuals with diabetes and they are known or presumed to affect health related quality of life. Sociodemographic characteristics included age (in years), gender, education (in years), current marital status (married, single/divorced/widow), comorbidities (self-reported conditions of heart attack, stroke, hypertension, visual deficit) and financial strain (none versus high). Financial strain was determined by asking subjects how much difficulty they have paying monthly bills ("a great deal, some, a little, or none") and how much money they usually have left over at the end of the month ("some money left over, just enough to make ends meet, or not enough to make ends meet"). Individuals were categorized as having financial strain if they responded that they either had a great deal of difficulty/some difficulty paying monthly bills, or if they had just enough/not enough money to make ends meet. Impaired vision was determined by asking subjects whether they could see well enough to recognize a friend (when wearing glasses/contacts if applicable) across the street, across the room, or at arm's length away. Choices to this question included yes, no, respondent is blind, or don't know. Responses were used to create a dichotomous variable, reflecting adequate vision or poor vision. If a subject was blind or answered no to any of the three conditions they were categorized as having poor vision.

Smoking status and body mass index (BMI) were also included as covariates. Smoking was categorized as ever smoked versus never smoked, and BMI was computed as weight in kilograms divided by height in meters squared. The presence of comorbidities was assessed by asking subjects if a physician had ever told them that they had cardiovascular problems (heart attack, myocardial infarction, or coronary thrombosis), stroke, or hypertension.

### Statistical Analysis

We compared baseline characteristics for the total sample and diabetes status (yes/no) using independent sample t-tests for continuous variables and chi-square statistics for categorical variables. A multivariate analysis of covariance (MANCOVA) was used to examine differences in SF-36 scores between individuals with diabetes and those without the disease. The model controlled for sociodemographic variables, comorbidities, and vision impairment, smoking status and BMI. We examined the homogeneity of within-group correlations by computing regression slopes for persons with and without diabetes for the covariates and SF-36 composite scale and also examined the linearity of the within group relationships between covariates and SF-36 composite scales. No statistically significant differences were found between the regression slopes for the two groups and the relationships among the continuous covariates and SF-36 composite scores were linear indicating assumption for the analyses were met. Our initial analyses examined the differences between persons with and without diabetes on the SF-36 physical and mental composite scales. We then conducted further analyses examining differences between the eight SF-36 domain scores for persons with and without diabetes. The p < .05 alpha level was used for individual statistical comparisons and the type 1 error rate was controlled using a modified Bonferonni analysis [33]. The Statistical Package for the Social Sciences (SPSS 14.0, Chicago, IL) was used for all statistical analyses.

## Results

The mean age of the 619 participants was 78.32 years (SD = 5.20), 59.6% were female, and 51.1% were married at the time of data collection. The overall prevalence of self-reported diabetes was 31.2%. The mean years of education was 5.15 (SD = 3.82) and more than three-quarters of the participants reported financial strain. Although more than half of the sample reported having hypertension, less than 5% reported previous heart attack or stroke. Demographic information for the entire sample and for persons with and without diabetes is presented in Table 1.

**Table 1:** Sociodemographic and Health-Related Characteristics for Sample

		Total		No Diabetes		Diabetes	
Characteristic		n	(%)	n	(%)	n	(%)
Sample	n	619	(100%)	426	(68.8%)	193	(31.2%)
Age in yrs *	mean (sd)	78.32	(5.20)	78.80	(5.46)	77.26	(4.40)
BMI in kg/m^2 ^†	mean (sd)	28.10	(5.42)	27.70	(5.16)	29.04	(5.89)
Education in yrs	mean (sd)	5.15	(3.82)	5.11	(3.73)	5.25	(4.00)
Sex	Women	369	(59.6%)	249	(58.5%)	120	(62.2%)
Married	Yes	316	(51.1%)	209	(49.1%)	107	(55.4%)
Financial Strain	High	476	(77.8%)	321	(76.1%)	155	(81.6%)
Heart Attack	Yes	29	(4.7%)	20	(4.7%)	9	(4.7%)
Stroke	Yes	23	(3.7%)	13	(3.1%)	10	(5.2%)
Hypertension *	Yes	336	(54.6%)	203	(48.0%)	133	(69.3%)
Vision	Poor	80	(12.9%)	52	(12.2%)	28	(14.5%)
Ever Smoked	Yes	267	(43.1%)	189	(44.4%)	78	(40.4%)

* = p < .001, † = p < .01: independent t-test and chi-square analyses for unadjusted comparisons between diabetes and no diabetes groups.

Responses missing for financial strain (n = 7), heart attack (n = 4), stroke (n = 3), and hypertension (n = 4).

Means for the physical and mental composite scales and the eight domains of the SF-36 are presented in Table 2. The lowest and highest ratings for persons with diabetes are on the physical functioning (mean = 51.12, SD = 32.02) and mental health (mean 80.40, SD = 16.40) subscales, respectively. Ratings for persons without diabetes ranged from a low mean of 62.75 (SD = 21.70) for the domain of general health perceptions to a high mean of 83.18 (SD = 22.75) for the domain of social functioning. Unadjusted univariate analyses demonstrated that the mean differences were larger for the four domains comprising the Physical Composite scale (physical functioning, role-physical, bodily pain, and general health,) than for the Mental Composite scale (vitality, social functioning, role-emotional and mental health).

**Table 2:** Mean (sd) of SF-36 Domain Ratings for Total Sample and Persons with and without Diabetes

	Total		No Diabetes		Diabetes		
Domain	Mean	(sd)	Mean	(sd)	Mean	(sd)	p-value*
PF	60.80	(33.26)	65.16	(32.92)	51.12	(32.02)	<.001
RP	67.30	(42.71)	71.76	(40.88)	57.42	(45.04)	<.001
BP	69.39	(25.80)	71.34	(25.52)	65.09	(25.97)	.005
GH	60.31	(21.96)	62.75	(21.70)	54.92	(21.61)	<.001
VT	65.07	(20.20)	66.56	(20.14)	61.78	(20.01)	.006
SF	81.56	(23.85)	83.18	(22.72)	77.99	(25.88)	.017
RE	81.22	(35.64)	82.75	(34.57)	77.81	(37.80)	.125
MH	80.82	(16.58)	81.01	(16.68)	80.40	(16.40)	.673
PCS	41.32	(12.62)	43.04	(12.22)	37.50	(12.69)	<.001
MCS	55.08	(8.53)	54.94	(8.15)	55.39	(9.32)	.566

PF = physical functioning, RP = role limitations due to physical functioning, BP = bodily pain, GH = general health, VT = vitality, SF = social functioning, RE = role limitations due to emotional problems, MH = mental health, PCS = standardized physical composite scale, MCS = standardized mental composite scale.

*p-values represent unadjusted (independent t-test) differences.

Results of the MANCOVA revealed statistically significant differences between persons with and without diabetes on the Physical Composite scale (F = 19.35, p < .001), but no statistically significant differences for the Mental Composite scale (F = 0.05, p = .821) controlling for age, sex, education, heart attack, stroke, hypertension, vision impairment, financial strain, ever smoked, and BMI (see Table 3). Since the overall multivariate test indicated a significant multivariate main effect for diabetes (Wilks' Lambda = .96, F = 2.82, df = 8, 535, p = .005), univariate probes were conducted across the eight SF-36 domains (see Table 3). Individuals with diabetes compared to those without diabetes displayed significantly (p < .05) lower scores on the physical functioning, role-physical, bodily pain, general health, vitality, and role-emotional subscales when controlling for sociodemographic variables and chronic illness. There were no statistically significant differences between persons with and without diabetes on the social functioning or mental health subscales of the SF-36.

**Table 3:** Between Groups MANCOVA Model Summary for the SF-36 Domains

Domains	MS	*df*	F	Sig.	95% CI
PF	14651.26	1	17.60	<.001	-17.11 to -6.20
RP	17094.78	1	10.87	.001	-20.08 to -5.09
BP	3617.18	1	6.42	.012	-10.28 to -1.30
GH	5084.74	1	12.43	<.001	-10.69 to -3.04
VT	1678.56	1	4.90	.027	-7.44 to -0.44
SF	957.90	1	2.13	.145	-6.99 to 1.03
RE	4399.90	1	3.94	.048	-12.71 to -0.06
MH	16.51	1	0.07	.792	-3.30 to 2.52
PCS	14651.26	1	17.60	<.001	-6.83 to -2.61
MCS	17094.78	1	10.87	.821	-1.34 to 1.69

Note: Diabetes is independent variable while controlling for age, BMI, sex, marital status, financial strain, heart attack, hypertension, vision impairment, and smoking.

Legend: PF = physical functioning, RP = role limitations due to physical functioning, BP = bodily pain, GH = general health, VT = vitality, SF = social functioning, RE = role limitations due to emotional problems, MH = mental health, PCS = standardized physical composite scale, MCS = standardized mental composite scale.

We repeated the MANCOVA analysis without the 20 participants identified as borderline diabetes (n = 173). The pattern of results in the SF-36 domain scores remained identical with one exception. The difference between vitality for persons with and without diabetes was no longer significant.

Regarding covariates, multivariate analyses revealed no significant association between the standardized composite scales and gender, marital status, education level, hypertension, or smoking history. Experiencing financial strain and visual impairment were each associated with lower scores on both the physical and mental composite scales. Age, BMI, and history of previous stroke were negatively associated with the Physical Composite scale, whereas previous heart attack demonstrated a negative relationship with the Mental Composite scale.

## Discussion

The purpose of this study was to assess the relationship between diabetes and health related quality of life in older Mexican Americans. We found significant differences in health related quality of life ratings as measured by the SF-36 between persons with and without diabetes. The differences in health related quality of life were largely associated with the physical domains of the SF-36 including physical functioning, role limitations due to physical functioning, bodily pain, and general health. We hypothesized that the SF-36 physical domains would be the most directly affected by the complications and pathology associated with diabetes. Morbidities related to diabetes, such as increased BMI, heart attack, stroke, and vision impairment were associated with poor health related quality of life, as were older age and reporting financial strain. Thus, it appears that overall health-related outcomes (i.e. physical, social, and emotional well-being) may be influenced by diabetes-related complications and compounded by an individual's physical and socioeconomic status [18,19,23]. It is also important to note that low physical component scores are robust predictors of death and hospitalization in community-dwelling older adults across all medical conditions [34].

After adjusting for age, sex, educational level, marital status, financial strain, heart attack, stroke, hypertension, smoking status, BMI and vision impairment, individuals with diabetes reported lower scores across several domains of the SF-36 – only social function and mental health subscales did not reach statistical significance (see Table 3). This finding is consistent with previous studies demonstrating that individuals with diabetes experience generally worse health than those without the disease [17,21,25,35,36]. In addition, low well-being among elderly patients with diabetes is an independent predictor of future health problems, including stroke [37]. Further study is necessary to determine if 1) interventions aimed at improving perceived quality of life can prevent the occurrence of more severe illness and/or 2) earlier diagnosis and better management can prevent diabetes-related complications and improve health related quality of life in older adults with diabetes.

There are multiple factors, besides the covariates included in this study that impact a person's health and are potentially responsible for the differences in the health related quality of life. Potential moderating or mediating variables include physical activity level, disease severity/glycemic control, and dietary habits [19,35]. We were limited in our analyses to those variables included in the Hispanic EPESE data set. In future research it will be important to identify additional potential correlates of health related quality of life and examine the extent to which those factors can be managed or changed [38]. Previous investigations have demonstrated, for example, that access to health care alone does not adequately explain the disparities exhibited by individuals with diabetes when compared to persons without diabetes [22].

Mean SF-36 domain ratings for persons in the diabetes group ranged from 51 to 80 and were generally higher than those reported in previous studies on health related quality of life and diabetes in other racial/ethnic groups [18,23,24]. One reason for this may be that the individuals in this investigation are part of a longitudinal study on aging, and the information analyzed here represents the fourth wave of data collected over a period of approximately nine years. Thus, participants who remained in the study likely represent individuals who have better general health compared to the original sample; i.e., they are the survivors. Conversely, these findings may reflect underlying ethnic characteristics associated with better overall perceptions of health in older Mexican Americans. Farley et al. [39] found that Mexican Americans reported better mental health functioning, based on SF-36 Mental Composite scale scores, than non-Hispanic Whites, despite no significant differences between groups in mean number of chronic health problems or perceived stress. These results suggest that Mexican Americans may tolerate health related stressors better than non-Hispanic Whites, and this may be related to the observed pattern of comparatively higher health related quality of ratings in this minority group. Peek et al., [27] for example, reported that health related quality of life ratings from a large sample of older Mexican Americans were higher than the national average. Similarly, Riegel et al. [40] found that health related quality of life improved more in Hispanics compared with non-Hispanics at six months following heart surgery, despite no significant differences between the groups initially.

'Older American' is not a homogeneous designation. The expectations and assumptions linked to aging vary by region, culture, individual, and circumstance. Certain qualities, such as increased life expectancy and multigenerational family support [5], are associated with certain minority groups, including U.S. Hispanics, and these qualities convey more favorable attitudes overall towards aging. Better stress-coping mechanisms have been postulated to possibly explain part of the Hispanic health paradox [39]; despite lower socio-economic status and reduced access to healthcare, U.S. Hispanics tend to experience lower morbidity and mortality compared with non-Hispanic whites [41]. It is also important to note that quality of life reflects the balance between expectations and experiences [42], both of which influence an individual's perceived status. Older Hispanics report lower expectations for health-related aspects of aging, including cognitive and mental health domains, relative to older African Americans and non-Hispanic whites [43]. Although we did not measure pre-existing optimism, lowered age-expectations may partially explain the lack of association between mental components of health related quality of life and disease status. This is an area that requires additional investigation.

There were several limitations to the current study. First, the analyses included cross-sectional data so we are unable to determine the direction of the relationship between diabetes and poor health related quality of life ratings. Second, this study relied on self-reported diabetes. It is possible that there were some individuals who had diabetes but were not diagnosed with the disease at the time of interview and, therefore, misclassified [10,25]. Clinical observation and medical records could provide a more precise diagnosis. The self-report approach, however, has been documented to provide reliable information and good agreement between self-reported and laboratory-confirmed diabetes, with overall agreements ranging from 94 to 99% across different ethnic cohorts [44-46].

Our investigation has several strengths including a large community-based sample, the combination of performance-based and self-reported measures of health, and the focus on Mexican Americans older adults – the fastest growing segment of the U.S. population over age 65 and a group that is exceedingly susceptible to diabetes and its complications. This population will consume an increasing proportion of health related services and resources in the coming decades. It is important that we increase our understanding of how health related quality of life and other health domains are affected by prevalent chronic conditions in this population that is becoming a more integral part of the U.S. healthcare landscape.

In summary, we found that Mexican American older adults with diabetes demonstrated reduced health related quality of life compared to persons without diabetes after adjusting for sociodemographic factors, physical characteristics and chronic health conditions. The reduction in perceived health related quality of life was primarily associated with the physical components of the SF-36. Continued research is needed to develop effective education, prevention and treatment programs to enhance health related quality of life for all older adults, but particular for underserved populations with chronic disease.

## Competing interests

The author(s) declare that they have no competing interests.

## Authors' contributions

JG wrote and revised drafts of manuscript, contributed to data analysis and interpretation of data. DS assisted in reviewing literature and preparing background information, participated in writing introduction, and interpreting data. GO contributed to concept and hypothesis development, interpretation of data and preparation of manuscript. SA conducted data analysis and assisted in interpretation and writing of methods section. MP conducted data analyses and interpretation, prepared code for data analysis, and assisted writing results. KM contributed to conceptual development, provided data, reviewed and revised sections of manuscript. KO participated in concept and hypothesis development, revising manuscript, and interpretation of data. All authors read and approved the final manuscript.
